# The Necessity of DNA Taxonomy to Reveal Cryptic Diversity and Spatial Distribution of Meiofauna, with a Focus on Nemertea

**DOI:** 10.1371/journal.pone.0104385

**Published:** 2014-08-05

**Authors:** Francesca Leasi, Jon L. Norenburg

**Affiliations:** Department of Invertebrate Zoology, Smithsonian National Museum of Natural History, Washington, District of Columbia, United States of America; University of Milan-Bicocca, Italy

## Abstract

Meiofauna represent one of the most abundant and diverse communities in marine benthic ecosystems. However, an accurate assessment of diversity at the level of species has been and remains challenging for these microscopic organisms. Therefore, for many taxa, especially the soft body forms such as nemerteans, which often lack clear diagnostic morphological traits, DNA taxonomy is an effective means to assess species diversity. Morphological taxonomy of Nemertea is well documented as complicated by scarcity of unambiguous character states and compromised by diagnoses of a majority of species (and higher clades) being inadequate or based on ambiguous characters and character states. Therefore, recent studies have advocated for the primacy of molecular tools to solve the taxonomy of this group. DNA taxonomy uncovers possible hidden cryptic species, provides a coherent means to systematize taxa in definite clades, and also reveals possible biogeographic patterns. Here, we analyze diversity of nemertean species by considering the barcode region of the mitochondrial gene Cytochrome Oxidase subunit I (COI) and different species delineation approaches in order to infer evolutionarily significant units. In the aim to uncover actual diversity of meiofaunal nemerteans across different sites in Central America, COI sequences were obtained for specimens assigned here to the genera *Cephalothrix, Ototyphlonemertes*, and *Tetrastemma*-like worms, each commonly encountered in our sampling. Additional genetic, taxonomic, and geographic data of other specimens belonging to these genera were added from GenBank. Results are consistent across different DNA taxonomy approaches, and revealed (i) the presence of several hidden cryptic species and (ii) numerous potential misidentifications due to traditional taxonomy. (iii) We additionally test a possible biogeographic pattern of taxonomic units revealed by this study, and, except for a few cases, the putative species seem not to be widely distributed, in contrast to what traditional taxonomy would suggest for the recognized morphotypes.

## Introduction

Reports of species occurrences and species lists are the basis for any biogeographic analysis. For meiofauna, which comprise interstitial benthic, often microscopic, animals, there are many problems in identifying ‘species’ as units of diversity. Moreover, this community often has been and remains overlooked because of taxonomic identification difficulties, and because the species were thought by some to be cosmopolitan; therefore, with no biogeographic interest [1], [2], [3]. Even in that context, meiofauna constitute among the most diverse, species-rich, and abundant communities of marine biocenoses; suites of organisms from many completely different evolutionary histories are present in the same habitat and in a relatively small sample at that [4]. This provides an invaluable model to identify generalities in macroecology and biogeography that transcend phylogenetic constraints [5].

Traditionally, meiofauna taxa, especially the soft-bodied forms (like gastrotrichs, platyhelminthes, polychaetes, etc.), are identified by morphological traits of living animals as soon as they are collected in the field [1], [4], [5], [6], [7]. Given the difficulties of finding reliable morphological taxonomic characters for most meiofauna, the putative widespread distributions of these organisms may be due to misidentification and lumping of cryptic species with restricted distributions [3], [8]. Nevertheless, with molecular approaches but even with higher resolution microscopy, complexes of cryptic species are reported from a broad systematic range of small marine animals, such as cycliophorans [9], copepods [10], [11], interstitial polychaetes [12], [13], platyhelminthes [14], [15], [16], rotifers [17], [18], nematodes [19], [20], [21], [22], gastrotrichs [23], and nemerteans [24], [25].

It has been established, and recently well supported, that the Cytochrome Oxidase subunit I (COI) identification system provides a reliable, cost-effective and accessible solution to the current problem of species identification, and can serve as the core of a global bioidentification system for animals [26], [27]. Hence, the recent application of COI sequences and molecular taxonomy approaches revealed actual taxonomic units of diversity and unexpected high levels of genetic differentiation with even higher degrees of cryptic diversity [27], [28], [29], [30], [31]. DNA taxonomy, apart from the pure discovery of hidden species diversity, may offer insights into the spatial structure of genetic diversity in understudied marine organisms and into the historical and ecological processes driving their present-day distribution. For meiofaunal organisms, which leave no fossil record, phylogeographic studies are the only possibility to get insights into such processes [32]. Detailed taxonomic investigations on these understudied organisms, by the use of DNA taxonomy approaches, suggested that ecological and/or geographical patterns of distribution may exist also for meiofaunal animals, contrary to a common idea that all are ubiquitous and cosmopolitan [33], [34]. On the other hand, this technique has not revealed a common biogeographic pattern for meiofauna. And, one might expect that ecological and/or spatial distribution at both local and large scales might be very different across different major clades and even within the same phylum. In some cases, molecular studies confirmed the existence of large-scale distributions despite lack of active means for dispersal, whereas other studies evidenced patterns of sympatric or parapatric speciation, in keeping with limited powers of dispersal [16], [30], [35]. According to Curini-Galletti et al. [5], meiofaunal groups with low dispersal potential have more restricted distributions and higher probabilities of harboring species new to science. They also argue dispersal ability as well as body size and habitat are crucial correlates of diversity for these understudied animals, with different importance at different spatial scales. To date, we have very little empirical evidence and understanding of the dispersal capability of most meiofaunal organisms. Consequently, biogeography of microscopic species remains controversial in current scientific discussion [3].

Here we present the results of a faunistic and taxonomic investigation of marine meiobenthic species belonging to the phylum Nemertea, which were collected from Belize, Caribbean Panama and Pacific Panama. Nemertea represents one of the most neglected groups in terms of estimating their diversity, because of a small number of specialists and also because apparent morphological complexity of its species is deceptive, making them among the trickiest organisms to identify morphologically. Differences between any of the species within meiofaunal genera may be very subtle [36], [37] but even in the presence of substantial discernible morphological variation, for instance within *Ototyphlonemertes*, the iconic genus of interstitial nemerteans, most of the variation is non-discrete [38] and there appears to be significant intrapopulational variation within sites (JLN, unpublished obs). Meiofaunal nemerteans, as other marine meiofauna, generally were considered to be widespread, without barriers to prevent gene flow among populations. However, a number of molecular studies suggested the likely presence of cryptic lineages [24], [25], [39], implying that biogeography of this group still needs to be well ascertained. In this context, we aim to disentangle nemertean diversity and compare traditional and molecular taxonomy. We examine the barcode region of the mitochondrial gene COI and use different species delimitation approaches to quantify the putative presence of cryptic species within marine meiofaunal Nemertea, and uncover, at least in part, their actual diversity. We sampled at the localities mentioned above because tropical diversity of many meiofaunal taxa, including nemerteans, is conspicuously understudied. Moreover, for coastal marine organisms genetic diversity generally increases with decreasing latitude [40], therefore, we expect to uncover a wide spectrum of diversity for this group. Additionally, we aim to test for potential differential effects of isolation by distance versus a physical geographic barrier – the Panama Isthmus – on gene flow between the Pacific and the Atlantic Oceans. We also consider diversity in a broader framework by adding information already available in GenBank.

## Material and Methods

### Sampling

**Figure 1:**
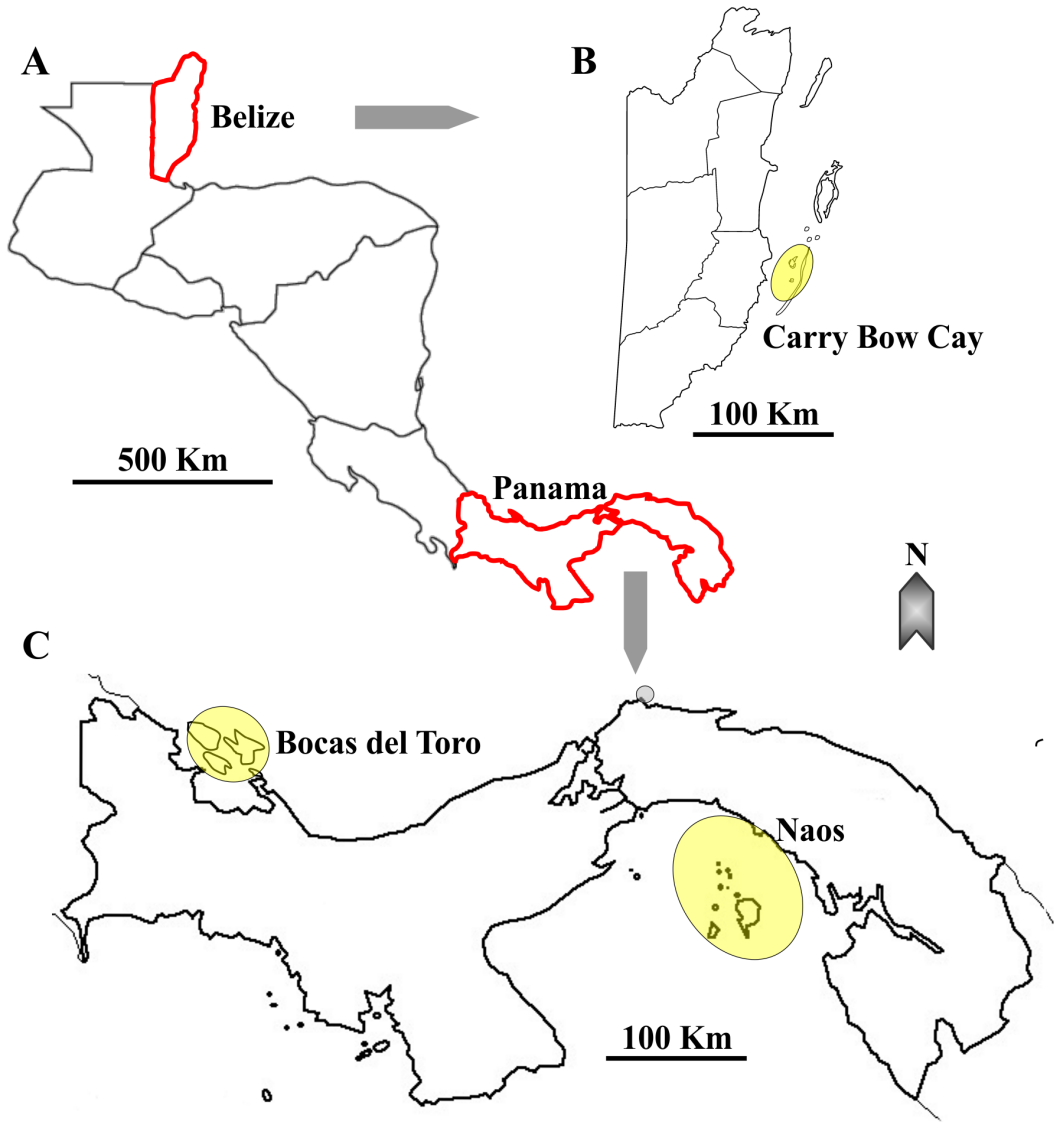
Map showing locations of sampling. A, view of Central America. Belize and Panama are indicated and their borders marked in red. B, close-up of Belize. The location of sampling, Carry Bow Cay, is indicated and highlighted within a yellow circle. C, Close-up of Panama. Bocas del Toro and Naos regions are each indicated and locations of sampling highlighted with a yellow circle.

Sediments were collected during three meiofauna workshops in sites located at Carrie Bow Cay in Belize (permit issued by James Azueta, Comptroller of Customs, Belize Fisheries Department, Ministry of Agriculture & Fisheries: Ref: GEN/FIS/15/04/10-52, Vol. III), and the vicinity of Bocas del Toro and Naos island, Panama (permits issued by Mario Quirós, Director General, Ecargado, Autoridad de los Recursos Acuáticos de Panama: Resolución DGOMI-PICFC-No. 40 de 31 Octubre de 2011), respectively in June 2010, October 2010, and December 2011 (Figure 1, Tables 1, 2, 3). No other sites were collected by us in this work and no endangered or protected species were involved in any of our work. Animals were extracted from the sediment using magnesium chloride isotonic to seawater, then isolated, identified to the lowest practical taxon rank, and transferred to 70% ethanol in DNA barcode tubes marked with unique extraction barcodes.

**Table 1:** Field stations at Belize where specimens of Nemertea were found.

Station	Locality	Depth (m)	Longitude	Latitude	Date (mm/dd/yy)
01	Carry Cay Bow, reef	10	16.8015	88.0790	01/14/10
02	Carry Cay Bow, reef	31	16.8037	88.0768	01/15/10
03	Carry Cay Bow, reef	15	16.8037	88.0768	01/16/10
04	Carry Cay Bow south	2–3	NA	NA	01/17/10
05	Carry Cay Bow	0	16.8025	88.0821	01/18/10
06	Carry Cay Bow, reef	3–5	16.8025	88.0798	01/19/10
07	Curlew Reef	2	16.7903	88.0787	01/19/10
08	Carry Cay Bow east	14	16.8021	88.0768	01/20/10
09	Carry Cay Bow, reef	30	16.8021	88.0768	01/20/10
10	Carry Cay Bow north	0	16.8025	88.0821	01/21/10
11	Carry Cay Bow south	0	16.8025	88.0821	01/21/10
12	Carry Cay Bow, reef	31	16.8021	88.0768	01/22/10
13	Carry Cay Bow south	0	NA	NA	01/23/10
14	Carry Cay Bow south	0	NA	NA	01/23/10
15	Carry Cay Bow south	0.1	NA	NA	01/23/10
16	Carry Cay Bow, reef	15	16.8021	88.0768	01/23/10
17	Carry Cay Bow, reef	42	16.8021	88.0768	01/23/10
18	Carry Cay Bow, reef	9–19	16.8024	88.0776	01/25/10

Depth, coordinates and date of sampling are indicated. NA =  not available.

**Table 2:** Field stations at Bocas del Toro where specimens of Nemertea were found.

Station	Locality	Depth (m)	Longitude	Latitude	Date (mm/dd/yy)
01	Smithsonian Marine Station	0	9.3609	82.2442	06/07/10
02	Bocas del Toro	0	9.3646	82.2479	06/16/10
03	Bird Rock	3	9.4583	82.3000	06/07/10
04	Solarte Garden	5	9.3222	82.2215	06/08/10
05	Wild Cane Rock	14	9.3502	82.1722	06/08/10
06	Wild Cane Reef	4	9.3449	82.1747	06/08/10
07	Crawl Cay Channel	NA	9.2521	82.1286	06/09/10
08	Tiger Rock	9	9.2140	81.9318	06/10/10
09	Peninsula Valiente	0	9.1476	81.9435	06/10/10
10	Wild Cane Reef	15	9.3506	82.1724	06/11/10
11	Wild Cane Cay	4	9.3473	82.1678	06/14/10
12	Boca del Drago Beach	NA	9.4160	82.3290	06/14/10
13	Wild Cane Key	NA	NA	NA	06/15/10
14	Wild Cane Reef	NA	9.3506	82.1724	06/15/10
15	Wild Cane Reef	3	9.3506	82.1724	06/11/10

Depth, coordinates and date of sampling are indicated. NA =  not available.

**Table 3:** Field stations at Naos region, Panama where specimens of Nemertea were found.

Station	Locality	Depth (m)	Longitude	Latitude	Date (mm/dd/yy)
01	Naos Island	0	8.9158	79.5305	12/01/11
02	Vera Cruz	0	8.8913	79.5954	12/01/11
03	Vera Cruz	0	8.8907	79.5951	12/02/11
04	Vera Cruz	0	8.8919	79.5953	12/01/11
05	Las Perlas	20	8.3878	79.1255	12/06/11
06	Las Perlas	0	8.3985	79.1175	12/06/11
07	Las Perlas	20	8.3927	79.1268	12/06/11
08	Taboga	9	8.8036	79.5540	12/09/11
09	Taboga	8	8.7823	79.5369	12/09/11
10	Taboga	0	8.8014	79.5543	12/09/11
11	Taboga	6–10	8.7823	79.5369	12/09/11
12	Taboga	0	8.8004	79.5545	12/09/11
13	Caribbean Sea	0–0.3	9.4702	79.7265	12/11/11
14	Isla Pacheco	15	8.6728	79.0609	12/12/11
15	Isla Pacheco	0	8.6728	79.0609	12/12/11
16	Isla Bartolome	0	8.6710	79.0648	12/12/11

Depth, coordinates and date of sampling are indicated.

### Amplification and sequencing

Genetic analyses of single individuals were done at the Laboratories of Analytical Biology, Smithsonian Institution. Tissue samples were digested with 150 µL of Autogen M2 buffer and 150 µL of Autogen M1 buffer with Proteinase K at 56°C in a shaker incubator. DNA extraction was performed using the Autogen Prep 956 Extractor. The DNA was eluted in 100 µL of Autogen R9 buffer. Polymerase Chain Reaction (PCR) was performed using a 15 ng template in a 50 ml volume (50 mM Tris–HCl pH 9.1, 16 mM (NH_4_)_2_SO_4_, 3.5 mM MgCl_2_, 150 mg ml^−1^ bovine serum albumin (BSA), 0.5 mM of each primer, 160 mM of each dNTP, and 0.25 ml of KlenTaq polymerase (AB Peptides, Inc.)). Thermo cycling comprised an initial 3-min denaturation at 95°C, followed by 40 cycles of 30 s at 95°C, 30 s at 48°C, 45 s at 72°C. The cycling ended with a 7-min sequence extension at 72°C. Amplification of parts of the coding region for COI was carried out using modified primers: dgLCO-1490 (CACGACGTTGTAAAACGACGGTCAACAAATCATAAAGAYATYGG) and dgHCO-2198 (GGATAACAATTTCACACAGGTAAACTTCAGGGTGACCAAARAAYCA) [41]. The PCR product was purified with QIAquick (Qiagen Inc.) and used in cycle sequencing with dye-terminators using BigDye chemistry (Perkin-Elmer) and standard cycles (4-min denaturation at 96°C, followed by 25 cycles of 10 s at 96°C, 5 s at 50°C and 4 min at 60°C), and sequenced on an ABI 3730xl 96-well capillary sequencer. The PCR primers were used for sequencing reactions.

### Alignment and phylogenetic inference

The sequences were first aligned using the ClustalW option implemented by Geneious v. 7.0.4 created by Biomatters (www.geneious.com). Additional COI sequences available in GenBank were added in order to better uncover diversity and, when possible, also a broader spatial distribution of taxa. Putative genera with a significant number of COI sequences (at least 30), obtained by a combination of our original data and GenBank, were considered and compared to each other in individual phylogenies. Our dataset comprises a total 370 COI sequences (Table S1): 191 of *Cephalothrix* spp. (Palaeonemertea, Cephalotrichidae), 72 of *Ototyphlonemertes* spp. (Enopla, Hoplonemertea, Monostilifera, Eumonostilifera, Ototyphlonemertidae), and 46 of cf. *Tetrastemma* spp. (Enopla, Hoplonemertea, Monostilifera, Eumonostilifera, Tetrastemmatidae; based solely on the presence of four ocelli and not intended to be phylogenetically meaningful). Though only some *Cephalothrix* spp., and a few *Tetrastemma*-like spp. [36], [37] traditionally are considered as meiofauna, because traditionally only interstitial nemerteans have been viewed with that lens, we encountered several presumed species of each in our sampling that fit a functional definition of meiofauna – able to pass through a 0.5-mm mesh sieve. Some are ‘typical’ interstitial forms from coarse sediments, others are merely small nemerteans from finer sediments that lack typical interstitial nemerteans, some may be psammophilic but more or less epibenthic (our sampling cannot distinguish), while others may represent temporary meiofauna (e.g., juveniles). We include them in our study because no objective *a priori* distinction is practical (e.g., for GenBank records size and ecological data normally are not available; we recognize that some, if correctly named, are not meiofaunal as adults and not likely to be found even as juveniles in traditional meiofaunal sampling). We reconstructed phylogenetic trees, with both maximum likelihood (ML) and Bayesian inference (BI). As outgroups for rooting, we used respectively the COI sequence of a species of *Tubulanus annulatus* (EU489497) [42], which is the paleonemertean sister taxon to *Cephalothrix* [24], [43]; *Ototyphlonemertes santacruzensis* (AJ436913) [44] was used as outgroup for *Tetrastemma*-like spp., and *Tetrastemma coronatum* (AY791975) [44] for *Ototyphlonemertes* spp., since both genera are Eumonostilifera.

The selected model of evolution for the phylogenetic reconstructions was general time-reversible-plus-gamma-distribution plus a proportion of invariant sites (GTR+I+G), chosen by hierarchical likelihood-ratio tests in ModelGenerator v. 2.145 [45]. This model was implemented into PhyML 3.0 [46] for the ML reconstruction, in which 1000 bootstrap replicates were used to evaluate node support. The same model was implemented in MrBayes 3.2.1 [47] for the BI reconstruction, in which we used two parallel runs and four independent Markov chains per run of five million generations; the first 25% of the trees were discarded to obtain the consensus tree. Ultrametric trees were generated using penalized likelihood in r8s [48] and cross-validation to choose the optimal smoothing parameter using the BI tree, which had very similar topology and support values to the ML tree.

We implemented four DNA taxonomy approaches to evaluate the presence of cryptic species. (1) The general mixed Yule-coalescent (GMYC) approach [49], [50] was applied to the ultrametric tree in R 2.15.3 [51] with the Splits package (http://splits.r-forge.r-project.org/). The GMYC model is a process-based approach for detecting the threshold in a gene tree at which within-species processes (i.e., coalescence) shift to between-species processes (i.e., speciation and extinction) [49], [50], [52]. (2) We applied the combination of the Poisson Tree Processes model for species delimitation (PTP), and a Bayesian implementation of PTP (bPTP) to infer putative species boundaries on a given phylogenetic input tree [53]. The PTP/bPTP model, unlike the GMYC model, requires a bifurcated phylogenetic tree, not an ultrametric tree [53]. PTP/dPTP models speciations or branching events in terms of number of substitutions. We used the following parameters: MCMC, 500000 generations; Thinning, 100; Burn-in, 0.1; Seed, 123, and always checked the convergence in order to be confident about the reliability of results. (3) We tested the consistency of the number of units of diversity obtained from both the GMYC and PTP models by looking for congruence with the results from Automatic Barcode Gap Discovery (ABGD) for primary species delimitation [54], and from (4) Nucleotide Divergence Threshold (NDT) analysis [26], applying a script written in R according to Tang et al. [27]. ABGD uses a range of prior intraspecific divergences to infer from the data a model-based one-sided confidence limit for interspecific divergence, whereas NDT is based on empirically observed gaps, with 97% being the most commonly used threshold for COI.

## Results

### Traditional taxonomy

Out of the total 222 specimens collected from 49 sites in the three major localities, a total of 67 morphotypes were designated. In Belize, nemerteans were found in 18 sites, with depth ranging from 0 to 42 m, a total of 88 individuals were assigned to 22 species ( =  morphotypes) and the following seven genera: *Annulonemertes*, *Cephalothrix*, *Hubrechtella*, *Nemertellina*, *Ototyphlonemertes*, *Poseidonemertes*, and cf. *Tetrastemma*. Station 12 (31 m depth) (Tables 1, 4) was the most diverse site in terms of number of species (n = 6). In Bocas del Toro, nemerteans were found in 15 sites with depth ranging from 0 to 15 m; a total of 70 individuals were collected and assigned to 19 morphotypes and the following six genera: *Annulonemertes*, *Cephalothrix*, *Hubrechtella*, *Ototyphlonemertes*, *Poseidonemertes*, and cf. *Tetrastemma*. Station 15 (3 m depth) (Tables 2, 5) was the most diverse, with six identified morphotypes. In the vicinity of Naos, we found meiobenthic nemerteans in 16 locations, with depth ranging from 0 to 20 m; a total of 64 individuals were collected and assigned to 26 morphological units and the following seven genera: *Carinomella*, *Cephalothrix*, *Hubrechtella*, *Nemertellina*, *Ototyphlonemertes*, *Riserius*, and cf. *Tetrastemma*. Station 16 (intertidal) (Table 3, 6) was the most diverse in terms of number of species (n = 10).

**Table 4:** List of taxa (22) found at different stations in Belize.

Taxon	Belize Station																	
	01	02	03	04	05	06	07	08	09	10	11	12	13	14	15	16	17	18
*Annulonemertes* sp.1												○						
***Cephalothrix alba***												**X**						
***Cephalothrix fasciculus***				**X**											**X**			
***Cephalothrix*** ** sp.1**									**X**									
***Cephalothrix*** ** sp.2**																	**X**	
***Cephalothrix*** ** sp.3**						**X**												
*Hubrechtella* sp.1		○																
Lineidae sp.1																		○
Lineidae sp.2		○																
Lineidae sp.3	○																	
Lineidae sp.4												○						
*Nemertellina* sp.1								○										
*Nemertellina* sp.2							○											
*Nemertellina* sp. 3																○		
***Ototyphlonemertes duplex***											**X**							**X**
***Ototyphlonemertes erneba***							**X**				**X**	**X**			**X**			
***Ototyphlonemertes lactea***			**X**		**X**					**X**	**X**		○	**X**	**X**			
***Ototyphlonemertes macintoshi***			**X**			**X**				**X**	**X**	**X**			**X**			
***Ototyphlonemertes santacruzensis***	**X**			**X**	○	**X**	**X**	○				**X**				**X**		
*Poseidonemertes* sp.1		○																
***Tetrastemma*** ** sp.1**	**X**																	
***Tetrastemma*** ** sp.2**												**X**						

Taxa for which COI sequence was used in this work are marked in bold. X =  sites where specimens used for molecular analyses where collected. ‘○’ =  sites where specimens were found but not used in the present genetic analysis.

**Table 5:** List of taxa (19) found at different stations in Bocas del Toro.

Taxon	Bocas del Toro Station														
	01	02	03	04	05	06	07	08	09	10	11	12	13	14	15
*Annulonemertes* sp.2					○	○									
*Cephalothrix* sp.4					○										
***Cephalothrix*** ** sp.5**													**X**		
***Cephalothrix*** ** sp.6**					**X**				**X**			**X**			X
***Cephalothrix*** ** sp.7**	**X**										**X**				**X**
*Cephalothrix* sp.8															○
Hoplonemertea sp.1															○
Hoplonemertea sp.2										○		○			
*Hubrechtella* sp.2				○							○				
Lineidae sp.5									○						
***Ototyphlonemertes duplex***	**X**	**X**	**X**	**X**			**X**	**X**							
***Ototyphlonemertes erneba***			**X**										**X**		**X**
***Ototyphlonemertes macintoshi***									**X**						
***Ototyphlonemertes santacruzensis***			**X**											**X**	**X**
*Ototyphlonemertes* sp.1		○													
*Poseidonemertes* sp.2							○								
***Tetrastemma*** ** sp.3**								**X**			**X**				
***Tetrastemma*** ** sp.4**					**X**										
*Tetrastemma* sp.5								○							

Taxa for which COI sequence was used in this work are marked in bold. X =  sites where specimens used for molecular analyses where collected. ‘○’ =  sites where specimens were found but not used in the present genetic analysis.

**Table 6:** List of taxa (26) found at different stations in Naos region.

Taxon	Naos Station															
	01	02	03	04	05	06	07	08	09	10	11	12	13	14	15	16
*Carinomella* sp.1									○							
***Cephalothrix alba***																**X**
*Cephalothrix linearis*									○			○				○
*Cephalothrix* sp.9				○		○										
***Cephalothrix*** ** sp.10**													**X**			
*Hubrechtella* sp.3											○					
Hubrechtidae sp.1					○											
Lineidae sp.6					○		○									
Lineidae sp.7																
Lineidae sp.8																○
Lineidae sp.9																○
Nemertea sp.1																○
Nemertea sp.2										○						
Nemertea sp.3								○								
Nemertea sp.4										○						○
Nemertea sp.5					○											
*Nemertellina* sp.4											○					
*Ototyphlonemertes cirrula*		○		○									○			
***Ototyphlonemertes duplex***		**X**	**X**	**X**												
***Ototyphlonemertes erneba***	**X**															**X**
***Ototyphlonemertes macintoshi***																**X**
***Ototyphlonemertes parmula***															**X**	**X**
*Paleonemertea* sp.1					○											
*Riserius* sp.1					○											
***Tetrastemma*** ** sp.6**														○		**X**
***Tetrastemma*** ** sp.7**								**X**								

Taxa for which COI sequence was used in this work are marked in bold. X =  sites where specimens used for molecular analyses where collected. ‘○’ =  sites where specimens were found but not used in the present genetic analysis.

### DNA taxonomy

**Figure 2:**
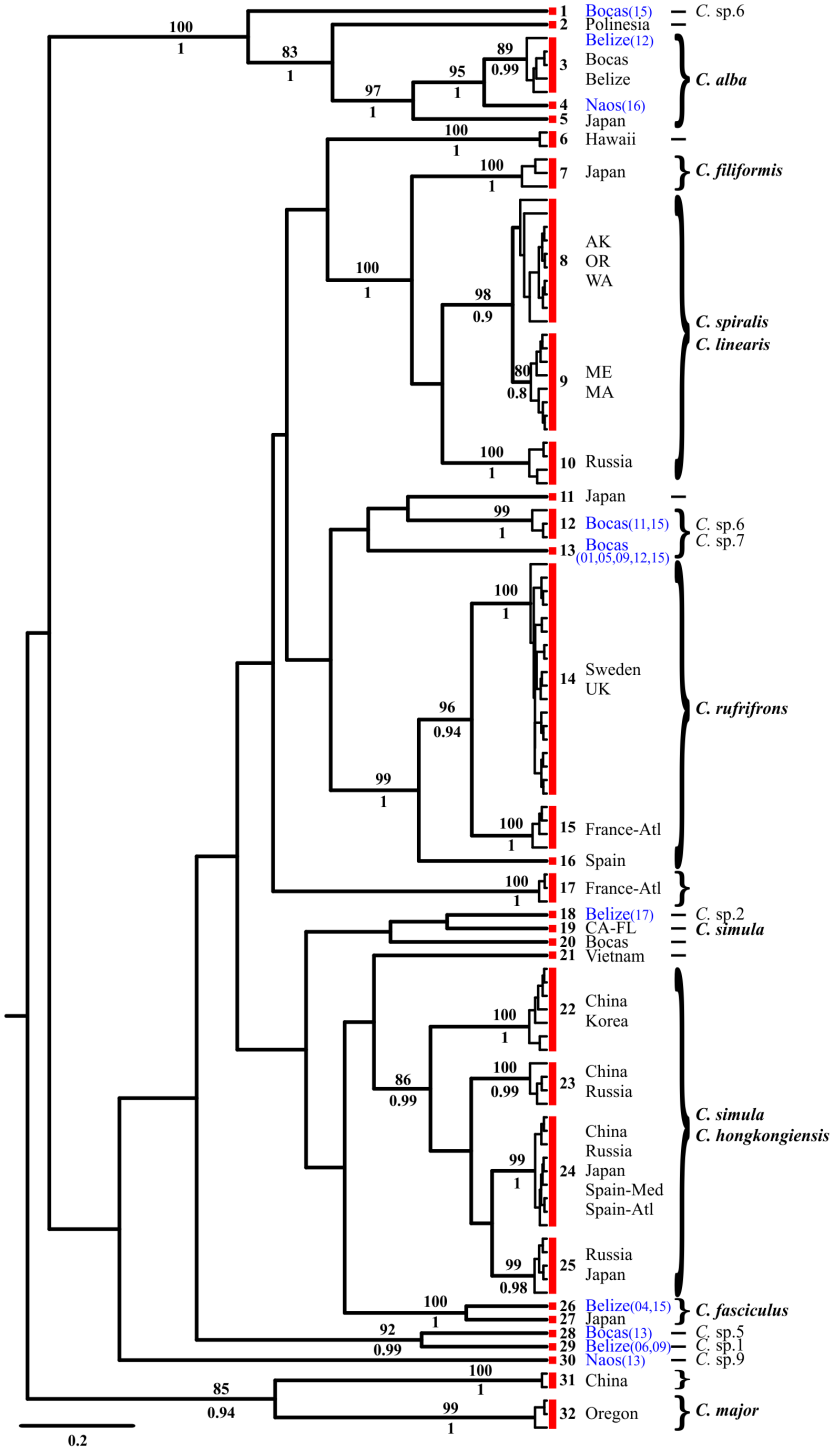
Phylogenetic relationships of the 106 cytochrome c oxidase subunit I (COI) haplotypes within the genus *Cephalothrix* spp. The consensus of 75,000 sampled trees from Bayesian analysis of the COI data sets is shown, displaying all compatible groupings, with average branch lengths proportional to numbers of substitutions per site under a general time reversible +I+G substitution model. Posterior probabilities from the Bayesian reconstruction and bootstrap support from the maximum likelihood reconstruction are shown below and above each branch, respectively. Support values are not shown for values below 0.8 for posterior probabilities, 80 for bootstrap support and for within-species short branches. Each species is indicated and grouped with a red box at left of tips, on its right the corresponding number of the entity (E.). Localities are shown and the ones where specimens for this work were collected are in blue, with the relative number of station between brackets. Morphotypes are presented on right. Unidentified species (*C*. spp.) are indicated only when specimens were collected for the present study.

**Figure 3:**
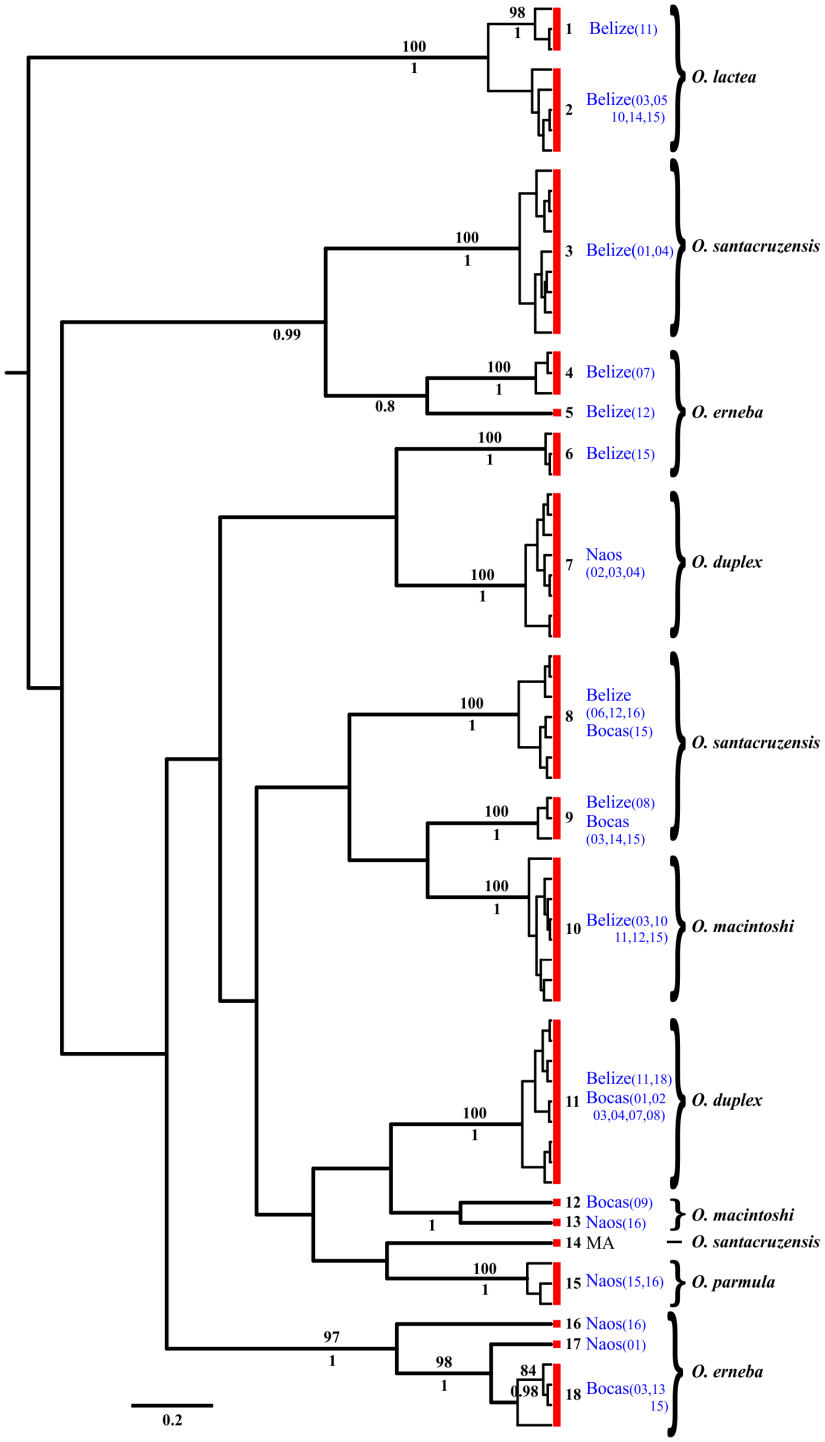
Phylogenetic relationships of the 70 cytochrome c oxidase subunit I (COI) haplotypes within the genus *Ototyphlonemertes* spp. The consensus of 75,000 sampled trees from Bayesian analysis of the COI data sets is shown, displaying all compatible groupings, with average branch lengths proportional to numbers of substitutions per site under a general time reversible +I+G substitution model. Posterior probabilities from the Bayesian reconstruction and bootstrap support from the maximum likelihood reconstruction are shown below and above each branch, respectively. Support values are not shown for values below 0.8 for posterior probabilities, 80 for bootstrap support and for within-species short branches. Each species is indicated and grouped with a red box at left of tips, on its right the relative number of the entity (E.). Localities are shown and the ones where specimens for this work were collected are in blue, with the relative number of station between brackets. Morphotypes are presented on right.

**Figure 4:**
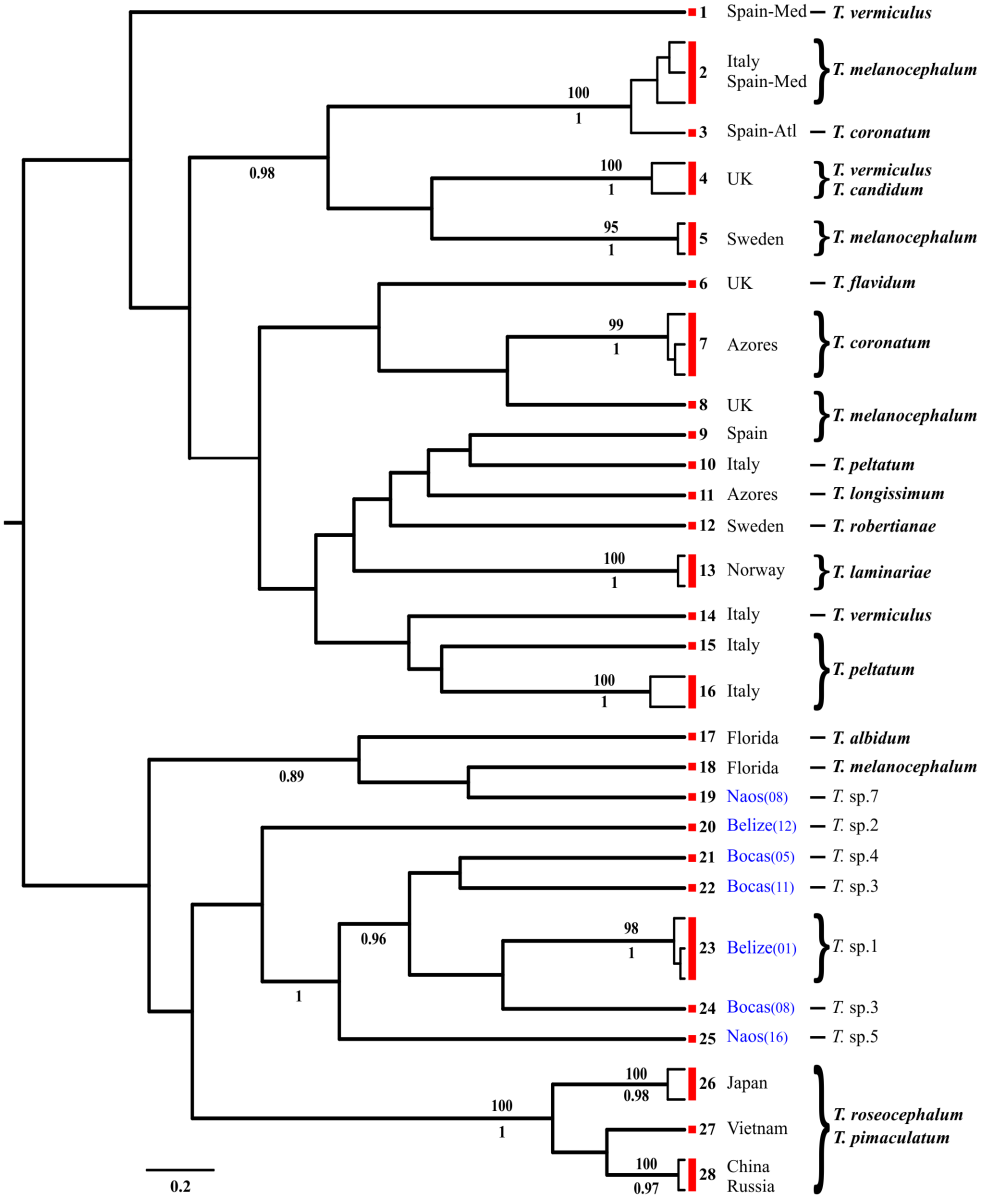
Phylogenetic relationships of the 40 cytochrome c oxidase subunit I (COI) haplotypes within the genus *Tetrastemma* spp. The consensus of 75,000 sampled trees from Bayesian analysis of the COI data sets is shown, displaying all compatible groupings, with average branch lengths proportional to numbers of substitutions per site under a general time reversible +I+G substitution model. Posterior probabilities from the Bayesian reconstruction and bootstrap support from the maximum likelihood reconstruction are shown below and above each branch, respectively. Support values are not shown for values below 0.8 for posterior probabilities, 80 for bootstrap support and for within-species short branches. Each species is indicated and grouped with a red box at left of tips, on its right the relative number of the entity (E.). Localities are shown and the ones where specimens for this work were collected are in blue, with the relative number of site between brackets. Morphotypes are presented on right. Unidentified species (*T.* spp.) are indicated only when specimens were collected for the present study.

We considered a total of 370 COI sequences obtained from specimens belonging to the genera *Cephalothrix*, *Ototyphlonemertes*, and cf. *Tetrastemma*; 72 individuals were from Belize, 55 from Bocas del Toro, and 28 from Naos (Tables 4, 5, 6). Within the genus *Cephalothrix*, we used 191 sequences, and out of the overall tree with 106 haplotypes, the GMYC model and PTP/bPTP approach suggested the presence of 32 entities as separate species (Figure 2; Table 7). The ABGD analysis yielded 31–34 groups, whereas NDT suggested the presence of 34 entities. Within *Ototyphlonemertes*, we obtained 72 sequences and 70 haplotypes. The GMYC model suggested the presence of 18 entities as separate species (Figure 3; Table 7). The PTP/bPTP approach recognized about 22–23 independent entities. The ML and Bayesian trees are slightly different from each other and from the GMYC tree. We here show results obtained with GMYC analysis, since they were supported by ABGD and NDT, which both suggested 18 taxonomic units (Figure 3; Table 7). For *Tetrastemma* spp., we obtained 46 sequences and 40 haplotypes. The GMYC model suggested the presence of 28 entities as separate species (Figure 4; Table 7). The PTP/bPTP approach recognized 29 independent entities, while the NDT and ABGD analyses both suggested the presence of 27 entities. Results obtained with GMYC analysis are shown in Figure 4.

**Table 7:** Number of entities (E.) for each analysed genus.

Taxon	#COI (#)	#H	#E.	GMYC	PTP/bPTP
*Cephalothrix* spp.	191 (11)	106	GMYC:32 ML:32 BI:32 ABGD:31–34 NDT:34	Likelihood null model: 704.4508; ikelihood best model: 736.1843; likelihood ratio: 63.46692; P-value <0.0001, confidence interval: 31–35	Acceptance rate: 0.147202; merge: 250229; split: 249771; estimated number species 31–41; mean: 32.71
*Ototyphlonemertes* spp.	72 (71)	70	GMYC:18 ML:16 BI:24 ABGD:18 NDT:18	Likelihood null model: 445.3766; likelihood best model: 456.4197; likelihood ratio: 22.08629; P-value <0.0001; confidence interval: 16–22	Acceptance rate: 0.343158; merge: 250194; split: 249806; estimated number species 16–35; mean: 22.45
*Tetrastemma* spp.	46 (8)	40	GMYC:28 ML:29 BI:29 ABGD:27 NDT:27	Likelihood null model: 178.3121; likelihood best model: 185.4611; likelihood ratio: 14.2981; P-value <0.001; confidence interval: 26–31	Acceptance rate: 0.152196; merge: 249681; split: 250319; estimated number species 25–34; mean: 28.76

Number of total COI sequences used (#COI), number of sequences obtained by this work (#),number of haplotypes (#H) are indicated for each genus. Number of entities (#E.) obtained by each analysis: GMYC model, ML and BI trees attained by PTP/bPTP approach, ABGD, NDT are shown. Moreover, outcomes parameters from GMYC and PTP/bPTP approaches are indicated in the last two columns.

Uncorrected genetic distances for COI within the putative cryptic species obtained by the GMYC model ranged from 0.15 to 2.13% in *Cephalothrix* spp. (mean ± standard deviation  = 0.60±0.46%), from 0.17 to 6.59% in *Ototyphlonemertes* spp. (mean ± standard deviation  = 1.37±1.33%), and from 0.17 to 1.86% in cf. *Tetrastemma* spp. (mean ± standard deviation  = 0.69±0.58%). Distances between them ranged from 1.06 to 36.36% (mean ± standard deviation  = 15.85±4.57%) in *Cephalothrix* spp., from 9.80 to 22.47% (mean ± standard deviation  = 17.17±2.20%) in *Ototyphlonemertes* spp., and from 1.67 to 20.59% (mean ± standard deviation  = 14.69±2.60%) in cf. *Tetrastemma* spp.

### Comparison between traditional and DNA taxonomy and geographical distribution

Among the morphotypes identified at the species level, within the genus *Cephalothrix* (Figure 2), *C. alba* morph appears to comprise at least three cryptic species ( =  entities, abbreviated with E.), one from the Atlantic side of Panama (comprising individuals from Bocas del Toro and Belize; E.3), one present in Pacific Panama (E.4), and the other from Japan (E.5). *Cephalothrix* cf. *alba*, in sizes ranging from meiofaunal to slightly thicker, is commonly associated (possibly preferentially) with tropical and subtropical sediments typically sampled for interstitial meiofauna (JLN, unpublished obs). *Cephalothrix simula* morph is a complex of at least five entities found in China and Korea (E.22), China and Russia (E.23), Russia and Japan (E.25), China, Russia, Japan, and Spain in both Atlantic and Mediterranean Sea (E.24), and USA with a single haplotype collected from sediments of both Florida and California (E.19). *Cephalothrix spiralis* morph investigated here comprises three entities found respectively in the White Sea, Russia (E.10), and the northwest (E.8) and northeast coasts of America (E.9), but some of our specimens resemble closely *C. spiralis* and *C. linearis*. Several potential but unsurprising taxonomic misidentifications are revealed; for instance, specimens identified as *C. linearis* share entity identity with *C. spiralis*, and *C. hongkongiensis* shares entity identity with *C. simula*. Within the genus *Ototyphlonemertes* (Figure 3), results obtained with the GMYC model show *O. lactea* morph comprising two cryptic species (E.1, E.2), both present in Belize but found in different stations (Figure 3). The *O*. *duplex* morph (diagnosed here by presence of two statolith granules) comprises two species: one from Pacific Panama (E.7) and one genetically disjunct species from Belize and Bocas del Toro (E.11). An *O*. *santacruzensis* morph (*O*. *pallida* morph *sensu* Envall and Norenburg [38], diagnosed here by presence usually of four statolith granules, but this varies from 2–8 within populations and even within specimens) comprises four species: one from Belize only (E.3), two shared by Belize and Bocas del Toro (hence, three species from Belize; E.8, E.9); each is genetically disjunct from a previously sequenced *O. santacruzensis* morph (AJ436913) from Massachusetts (E.14). An *O*. *erneba* morph (diagnosed here by presence of three statolith granules, stylet to basis ratio >2, and body wall dissolving in MgCl_2_) comprises at least six species: three from Belize (E.4, E.5, E.6), one for Bocas del Toro (E.18), plus a couple of outlier entities from Pacific Panama (E.16, E.17), which are genetically close to the ones of Bocas del Toro. The latter clade, comprising entities from Naos and Bocas del Toro is well supported by high bootstrap and posterior probability values (Figure 3). An *O*. *macintoshi* morph (diagnosed here by polygranular statolith and proboscis with tubular middle chamber) is represented here by perhaps three species: one in Belize (E.10), one from Bocas del Toro (E.12), and one from Pacific Panama (E.13). The *Tetrastemma*-like group (Figure 4) reveals five cryptic species in *T. melanocephalum* morph, with at least one entity found in the Mediterranean Sea (E.2), northern Europe (E.5), UK (E.8), Spain (E.9), and Florida (E.18). GenBank sequences considered here for *T. roseocephalum* morph and *T. pimaculatum* morph appear to represent a single clade, comprising at least three cryptic species (respectively from Japan, Vietnam, and China: E.26, E.27, E.28). Also, GenBank sequences recorded as *T. vermiculus* comprised several entities from the Mediterreanean Sea (E.1, E.14) and UK (E.4), whereas those assigned to *T. peltatum* morph showed three Italian entities (E.10, E.15, E.16).

Most entities investigated in this work, whether meiofaunal or of unknown size, were confined to a particular geographical area or a single ocean. Only three entities might be considered cosmopolitan because they are distributed among disjunct oceans: 1) a species of *C. simula* morph, which was found in Japan, East Atlantic Ocean, and Mediterranean Sea [55], 2) a *Cephalothrix* identified as *C. simula* but not in the cluster assigned to that species [56], with the same haplotype found once in both Florida and California, and 3) a *Tetrastemma* found in East Atlantic Ocean and Mediterranean Sea.

## Discussion and Conclusion

This work supports the importance of combining genetic and morphological information in order to disentangle the actual diversity of meiofaunal organisms. The ‘barcoding region’ of the COI gene seems able to resolve species identity in nemerteans, revealing a degree of cryptic speciation comparable to other meiofauna taxa investigated so far, and shows cases of likely morphological misidentification. Moreover, our data are not able to strongly support previous assumptions for cosmopolitan distributions of nemerteans.

### Cryptic diversity and comparison between traditional and DNA taxonomy

Finding cryptic lineages is not surprising in light of several studies on nemerteans showing lack of concordance between morphological and molecular diversity [39], [44], [56], [57], [58], [59]. However, to date DNA taxonomy in nemerteans has been restricted to statistical parsimony [56] and not the species delineation approaches used here. According to our results, DNA taxonomy provides a higher diversity than traditional taxonomy, with a barcoding gap in nemerteans comparable to other meiofauna groups. For instance, within the rotifer *Brachionus plicatilis* and *Testudinella clypeata* complexes, the average uncorrected pairwise COI distances are respectively 3.4 and 2.7% within entities, and 18.9 and 20.8% between them [30], [33]. Different gastrotrich morphotypes revealed an average pairwise COI distance of 0.5–8.1% within entities and 25–38% between them [34].

In the present work at least 26 morphotypes were identified and assigned to a named species morphotype, whereas a number of other individuals were assigned to higher taxon ranks. Within these 26 recognized species, 10 species belong to the genus *Cephalothrix*, six to *Ototyphlonemertes*, and 10 species are *Tetrastemma*-like. DNA taxonomy for the COI sequence of these particular individuals revealed actually a total of 58 entities (18 *Cephalothrix*, 18 *Ototyphlonemertes*, and 22 cf. *Tetrastemma*). This might be expected, since cryptic lineages were already suggested within all these genera [24], [25], [39], [44], [45], [56]. For instance, the *O. lactea* morphotype was already suggested to be a putative complex of cryptic species by Andrade et al. [24], whereas Tulchinsky et al. [25] suggested the presence of several cryptic lineages within *O. parmula*. The genus *Ototyphlonemertes* does not possess unambiguous diagnostic morphological traits across different species, which is why Envall & Norenburg [38] reduced the known species and about 75 additional regional varieties to six so-called phylomorphs. DNA taxonomy also facilitates improved systematization of some taxa that were not morphologically recognized at the level of species. For examples, within *Cephalothrix* spp., one unidentified species from Japan and one from Russia (GU726661, GU726641) may be finally considered as *C. simula*. Mostly, molecular taxonomy uncovers failures in species identification obtained by traditional taxonomy because of (i) possible human mistakes, (ii) incorrect use of morphological traits, or (iii) unpredictable presence of cryptic species. This corroborates recent assertions about the lack of reliable morphological traits to identify nemerteans at the species level, and that today DNA taxonomy is essential to estimate the actual diversity of meiofaunal and other nemerteans. The same outcome was suggested by Strand & Sundberg [44] and Fernández-Álvarez & Machordom [39], who found little or no correspondence between evolutionary lineage and morphotype for *Tetrastemma* and *Cephalothrix* investigated by them. The relatively high ratio of entities to haplotypes for *Tetrastemma*-like forms found here might be due to a taxon- and/or sample bias, or most experts would recognize it as the likely result of ours being a small sampling of a very large and cladistically heterogeneous universe of four-eyed distromatonemerteans [43]. In conclusion, our results confirm that morphological species delimitation in nemerteans should always be questioned, and that DNA taxonomy may have a profound effect upon estimates of species diversity within the phylum. Therefore, nemertean taxonomy likely depends on genotyping as a first step in identifications, as also advocated by Strand & Sundberg [60].

### Spatial structure of genetic diversity

Microscopic and meiofaunal organisms generally are believed to be cosmopolitan and one might infer that this should be true for meiofaunal nemerteans. A couple of molecular works, mostly based on the use of CO3, revealed a relatively wide-spread distribution of some *Ototyphlonemertes* species, without any apparent eco/physical barrier to prevent gene flow among populations, while simultaneously discovering more narrowly distributed cryptic species [24], [25]. It was suggested that a low genetic structuring in *O. parmula*, despite apparent limited dispersal potential, may be explained by infrequent long-distance dispersal of adults combined with a high colonization success rate [25]. Chen et al. [56] recently revealed, by using COI and statistical parsimony networks, a stronger biogeographic pattern among species of *Cephalothrix*. This might argue that the barcode region of COI better estimates diversity, compared to CO3, and is more suitable to delineate species, and uncover possible biogeographic patterns within this group of organisms. However, biogeographic distribution might be different in the two taxa. In the present study we are not able to uncover a comparable spatial scale in the two genera, since the only COI sequence of *Ototyphlonemertes* in GenBank relevant to our study is from Massachusetts. Therefore, we expect to investigate more deeply the phylogeography of *Ototyphlonemertes* by using COI at a larger spatial scale in order to solve this puzzle, and better understand distribution and diversity of this group.

In the present work only three entities might be considered truly cosmopolitan, because they are distributed among disjunct oceans: one entity of *C. simula*, occurring in Japan, East Atlantic, and Mediterranean Sea, which was already suggested as an artificial introduction via ballast water, or ship-fouling communities, or the commercially cultured oyster brought from Japan to France in 1970s [55]. In addition, as already indicated by Chen et al. [56], a shared haplotype belonging to the morphotype *C. simula* species complex has been reported for both California and Florida. Additionally, *T. melanocephalum* is recorded from the eastern Atlantic Ocean and the Mediterranean Sea. Nevertheless, most entities of the three genera are relatively confined in space and some morphotypes, which could be considered widely distributed, comprise cryptic species complexes, each lineage with a relatively limited distribution. In particular, the *C. spiralis* species complex comprises at least three different cryptic species, one present along the northeast coast of USA (Maine, Massachusetts), one along the northwest coast (Alaska, Oregon, Washington state), and the third one in the White Sea, Russia. *Ototyphlonemertes erneba* morph, *O. duplex* morph and *O. macintoshi* morph were morphologically identified from Pacific and Caribbean sites; however the respective putative populations seem not to encompass the same entities. Therefore, the American Continent might actually represent a physical barrier to the dispersion of these animals.

In conclusion, there is no obvious difference in distribution patterns between our own definitive meiofaunal nemerteans and the nemerteans selected from GenBank. Our results suggest that nemerteans show a very high genetic diversity and no clear inference could be performed with the available data. Nonetheless, the same uncertainties of possible spatial structure, coupled with occasional evidence of long-distance dispersal constrain similar past studies of marine nematodes [21], copepods [61], and gastrotrichs [34]. However, at present it is difficult to infer whether this combination of patterns could be due to (i) natural long/short distance dispersions, or (ii) to human/animal-mediated dispersal, or (iii) to sampling effort that affects our awareness of diversity and distribution of these organisms. Only with improved taxon- and location sampling combined with the use of other genes might we better elucidate the actual distribution of these tiny organisms, their dispersion in presence of potential barriers to their gene flow, and the correlation between their biogeography and their own peculiar biology, ecology, and evolutionary history.

## Supporting Information

Table S1 **Morphotype, cryptic species (number with prefix E.), Locality, GenBank accession number, and relative bibliographic reference are indicated for each COI sequence of** ***Cephalothrix*** **spp.,** ***Ototyphlonemertes*** **spp., and** ***Tetrastemma*** **spp. used in the present work.** Specimens are ordered by entity.
(DOCX)
