# Supplementary material for: The Necessity of DNA Taxonomy to Reveal Cryptic Diversity and Spatial Distribution of Meiofauna, with a Focus on Nemertea
Source: PLoS One. 2014 Aug 5;9(8):e104385. doi: 10.1371/journal.pone.0104385 (PMC4122443; doi:10.1371/journal.pone.0104385)
Supplement: Table S1 — Morphotype, cryptic species (number with prefix E.), Locality, GenBank accession number, and relative bibliographic reference are indicated for each COI sequence of Cephalothrix spp., Ototyphlonemertes spp., and Tetrastemma spp. used in the present work. Specimens are ordered by entity. (DOCX) [file pone.0104385.s001.docx]

| **Morphotype** | **Entity** | **Locality** | **GenBank #** | **Reference** |
| --- | --- | --- | --- | --- |
|  |  |  |  |  |
| ***Cephalothrix* spp.** |  |  |  |  |
| *C* sp. | E.1 | Bocas del Toro | KM083820 | Present study |
| *C* sp. | E.2 | French Polinesia | KC706830 | Leray et al. 2013 |
| *C.* alba | E.3 | Bocas del Toro | GU726677 | Chen et al. 2010 |
| *C.* alba | E.3 | Bocas del Toro | GU726679 | Chen et al. 2010 |
| *C.* alba | E.3 | Belize | GU726682 | Chen et al. 2010 |
| *C.* alba | E.3 | Belize | KM083817 | Present study |
| *C.* alba | E.3 | Belize | KM083818 | Present study |
| *C.* alba | E.4 | Naos | KM083819 | Present study |
| *C.* alba | E.5 | Japan | GU726666 | Chen et al. 2010 |
| *C* sp. | E.6 | Hawaii | GU726633 | Chen et al. 2010 |
| *C* sp. | E.6 | Hawaii | GU726634 | Chen et al. 2010 |
| *C. filiformis* | E.7 | Japan | GU726635 | Chen et al. 2010 |
| *C. filiformis* | E.7 | Japan | GU726637 | Chen et al. 2010 |
| *C. filiformis* | E.7 | Japan | GU726645 | Chen et al. 2010 |
| *C spiralis* | E.8 | USA, WA | GU726648 | Chen et al. 2010 |
| *C spiralis* | E.8 | USA, AK | GU726709 | Chen et al. 2010 |
| *C spiralis* | E.8 | USA, OR | GU726692 | Chen et al. 2010 |
| *C spiralis* | E.8 | USA, OR | GU726693 | Chen et al. 2010 |
| *C spiralis* | E.8 | USA, OR | GU726694 | Chen et al. 2010 |
| *C spiralis* | E.8 | USA, OR | GU726695 | Chen et al. 2010 |
| *C spiralis* | E.8 | USA, OR | GU726696 | Chen et al. 2010 |
| *C spiralis* | E.8 | USA, AK | GU726712 | Chen et al. 2010 |
| *C spiralis* | E.8 | USA, AK | GU726710 | Chen et al. 2010 |
| *C spiralis* | E.8 | USA, AK | GU726711 | Chen et al. 2010 |
| *C spiralis* | E.9 | USA, ME | GU726699 | Chen et al. 2010 |
| *C spiralis* | E.9 | USA, ME | GU726701 | Chen et al. 2010 |
| *C spiralis* | E.9 | USA, ME | GU726703 | Chen et al. 2010 |
| *C spiralis* | E.9 | USA, ME | GU726706 | Chen et al. 2010 |
| *C spiralis* | E.9 | USA, MA | GU726707 | Chen et al. 2010 |
| *C spiralis* | E.9 | USA, MA | GU726708 | Chen et al. 2010 |
| *C spiralis* | E.9 | USA, ME | GU726698 | Chen et al. 2010 |
| *C spiralis* | E.9 | USA, ME | GU726697 | Chen et al. 2010 |
| *C. linearis* | E.10 | Russia | GU726652 | Chen et al. 2010 |
| *C. linearis* | E.10 | Russia | GU726653 | Chen et al. 2010 |
| *C. spiralis* | E.10 | Russia | GU726649 | Chen et al. 2010 |
| *C. spiralis* | E.10 | Russia | GU726650 | Chen et al. 2010 |
| *C* sp. | E.11 | Japan | GU726667 | Chen et al. 2010 |
| *C* sp. | E.12 | Bocas del Toro | GU726680 | Chen et al. 2010 |
| *C* sp. | E.12 | Bocas del Toro | KM083809 | Present study |
| *C* sp. | E.12 | Bocas del Toro | KM083810 | Present study |
| *C* sp. | E.13 | Bocas del Toro | KM083811 | Present study |
| *C. rufifrons* | E.14 | Sweden | GU726593 | Chen et al. 2010 |
| *C. rufifrons* | E.14 | UK | GU726632 | Chen et al. 2010 |
| *C. rufifrons* | E.14 | Sweden | GU726688 | Chen et al. 2010 |
| *C. rufifrons* | E.14 | UK | GU726601 | Chen et al. 2010 |
| *C. rufifrons* | E.14 | Sweden | GU726598 | Chen et al. 2010 |
| *C. rufifrons* | E.14 | Sweden | GU726594 | Chen et al. 2010 |
| *C. rufifrons* | E.14 | Sweden | GU726591 | Chen et al. 2010 |
| *C. rufifrons* | E.14 | Sweden | GU726590 | Chen et al. 2010 |
| *C. rufifrons* | E.14 | Sweden | GU726592 | Chen et al. 2010 |
| *C. rufifrons* | E.14 | Sweden | GU726596 | Chen et al. 2010 |
| *C. rufifrons* | E.14 | UK | GU726603 | Chen et al. 2010 |
| *C. rufifrons* | E.14 | UK | GU726602 | Chen et al. 2010 |
| *C. rufifrons* | E.14 | UK | GU726604 | Chen et al. 2010 |
| *C. rufifrons* | E.14 | Sweden | GU726599 | Chen et al. 2010 |
| *C. rufifrons* | E.14 | Sweden | GU726597 | Chen et al. 2010 |
| *C. rufifrons* | E.14 | Sweden | GU726605 | Chen et al. 2010 |
| *C. rufifrons* | E.14 | Sweden | GU726728 | Chen et al. 2010 |
| *C. rufifrons* | E.14 | Sweden | GU726713 | Chen et al. 2010 |
| *C. rufifrons* | E.15 | France, Roscoff | GU726673 | Chen et al. 2010 |
| *C. rufifrons* | E.15 | France, Roscoff | GU726674 | Chen et al. 2010 |
| *C. rufifrons* | E.15 | France, Roscoff | GU726675 | Chen et al. 2010 |
| *C. rufifrons* | E.15 | France, Roscoff | GU726676 | Chen et al. 2010 |
| *C. rufifrons* | E.16 | Spain | GU726616 | Chen et al. 2010 |
| *C* sp. | E.17 | France, Roscoff | GU726670 | Chen et al. 2010 |
| *C* sp. | E.17 | France, Roscoff | GU726671 | Chen et al. 2010 |
| *C* sp. | E.17 | France, Roscoff | GU726672 | Chen et al. 2010 |
| *C* sp. | E.18 | Belize | KM083815 | Present study |
| *C. simula* | E.19 | USA, CA | GU726640 | Chen et al. 2010 |
| *C* sp. | E.20 | Bocas del Toro | GU726681 | Chen et al. 2010 |
| *C* sp. | E.21 | Vietnam | GU726621 | Chen et al. 2010 |
| *C. hongkongiensis* | E.22 | China | GU726610 | Chen et al. 2010 |
| *C. hongkongiensis* | E.22 | China | GU726612 | Chen et al. 2010 |
| *C. hongkongiensis* | E.22 | China | GU726613 | Chen et al. 2010 |
| *C.* sp. | E.22 | China | GU726614 | Chen et al. 2010 |
| *C. simula* | E.22 | China | GU726628 | Chen et al. 2010 |
| *C.* sp. | E.22 | Korea | GU726644 | Chen et al. 2010 |
| *C.* sp. | E.22 | China | GU726617 | Chen et al. 2010 |
| *C. simula* | E.23 | Russia | GU726607 | Chen et al. 2010 |
| *C.* sp. | E.23 | China | GU726618 | Chen et al. 2010 |
| *C.* sp. | E.23 | Russia | GU726608 | Chen et al. 2010 |
| *C.* sp. | E.23 | China | GU726624 | Chen et al. 2010 |
| *C. simula* | E.24 | Spain, Atl | JX453468 | Fernández-Álvarez, Machordom 2013 |
| *C. simula* | E.24 | Spain, Med | JX453470 | Fernández-Álvarez, Machordom 2013 |
| *C. simula* | E.24 | Spain, Med | JX453475 | Fernández-Álvarez, Machordom 2013 |
| *C. simula* | E.24 | Spain, Med | JX453488 | Fernández-Álvarez, Machordom 2013 |
| *C.* sp. | E.24 | Japan | GU726620 | Chen et al. 2010 |
| *C* sp. | E.24 | Japan | GU726664 | Chen et al. 2010 |
| *C* sp. | E.24 | Japan | GU726661 | Chen et al. 2010 |
| *C* sp. | E.24 | China | GU726615 | Chen et al. 2010 |
| *C* sp. | E.24 | Japan | GU726622 | Chen et al. 2010 |
| *C. simula* | E.25 | Russia | GU726609 | Chen et al. 2010 |
| *C. simula* | E.25 | Japan | GU726642 | Chen et al. 2010 |
| *C* sp. | E.25 | Russia | GU726641 | Chen et al. 2010 |
| *C* sp. | E.25 | Japan | GU726662 | Chen et al. 2010 |
| *C* sp. | E.25 | Japan | GU726663 | Chen et al. 2010 |
| *C* sp. | E.26 | Belize | KM083814 | Present study |
| *C. fasciculus* | E.27 | Japan | GU726623 | Chen et al. 2010 |
| *C* sp. | E.28 | Bocas del Toro | KM083812 | Present study |
| *C* sp. | E.29 | Belize | KM083813 | Present study |
| *C* sp. | E.30 | Panama | KM083816 | Present study |
| *C* sp. | E.31 | China | GU726629 | Chen et al. 2010 |
| *C* sp. | E.31 | China | GU726630 | Chen et al. 2010 |
| *C. major* | E.32 | USA, OR | GU726689 | Chen et al. 2010 |
| *C. major* | E.32 | USA, OR | GU726690 | Chen et al. 2010 |
| *C. major* | E.32 | USA, OR | GU726691 | Chen et al. 2010 |
|  |  |  |  |  |
| ***Ototyphlonemertes spp.*** |  |  |  |  |
| *O. lactea* | E.1 | Belize | KM083840 | Present study |
| *O. lactea* | E.1 | Belize | KM083863 | Present study |
| *O. lactea* | E.1 | Belize | KM083874 | Present study |
| *O. lactea* | E.2 | Belize | KM083826 | Present study |
| *O. lactea* | E.2 | Belize | KM083836 | Present study |
| *O. lactea* | E.2 | Belize | KM083852 | Present study |
| *O. lactea* | E.2 | Belize | KM083864 | Present study |
| *O. lactea* | E.2 | Belize | KM083888 | Present study |
| *O. santacruzensis* | E.3 | Belize | KM083822 | Present study |
| *O. santacruzensis* | E.3 | Belize | KM083851 | Present study |
| *O. santacruzensis* | E.3 | Belize | KM083860 | Present study |
| *O. santacruzensis* | E.3 | Belize | KM083868 | Present study |
| *O. santacruzensis* | E.3 | Belize | KM083872 | Present study |
| *O. santacruzensis* | E.3 | Belize | KM083879 | Present study |
| *O. santacruzensis* | E.3 | Belize | KM083881 | Present study |
| *O. santacruzensis* | E.3 | Belize | KM083883 | Present study |
| *O. santacruzensis* | E.3 | Belize | KM083886 | Present study |
| *O. erneba* | E.4 | Belize | KM083844 | Present study |
| *O. erneba* | E.4 | Belize | KM083873 | Present study |
| *O. erneba* | E.4 | Belize | KM083882 | Present study |
| *O. erneba* | E.5 | Belize | KM083858 | Present study |
| *O. erneba* | E.6 | Belize | KM083845 | Present study |
| *O. erneba* | E.6 | Belize | KM083853 | Present study |
| *O. erneba* | E.6 | Belize | KM083862 | Present study |
| *O. duplex* | E.7 | Naos | KM083823 | Present study |
| *O. duplex* | E.7 | Naos | KM083824 | Present study |
| *O. duplex* | E.7 | Naos | KM083827 | Present study |
| *O. duplex* | E.7 | Naos | KM083829 | Present study |
| *O. duplex* | E.7 | Naos | KM083831 | Present study |
| *O. duplex* | E.7 | Naos | KM083832 | Present study |
| *O. duplex* | E.7 | Naos | KM083833 | Present study |
| *O. duplex* | E.7 | Naos | KM083834 | Present study |
| *O. santacruzensis* | E.8 | Belize | KM083825 | Present study |
| *O. santacruzensis* | E.8 | Belize | KM083830 | Present study |
| *O. santacruzensis* | E.8 | Belize | KM083839 | Present study |
| *O. santacruzensis* | E.8 | Bocas del Toro | KM083843 | Present study |
| *O. santacruzensis* | E.8 | Bocas del Toro | KM083865 | Present study |
| *O. santacruzensis* | E.8 | Bocas del Toro | KM083880 | Present study |
| *O. santacruzensis* | E.8 | Bocas del Toro | KM083887 | Present study |
| *O. santacruzensis* | E.9 | Bocas del Toro | KM083821 | Present study |
| *O. santacruzensis* | E.9 | Bocas del Toro | KM083835 | Present study |
| *O. santacruzensis* | E.9 | Belize | KM083849 | Present study |
| *O. macintoshi* | E.10 | Belize | KM083828 | Present study |
| *O. macintoshi* | E.10 | Belize | KM083838 | Present study |
| *O. macintoshi* | E.10 | Belize | KM083837 | Present study |
| *O. macintoshi* | E.10 | Belize | KM083841 | Present study |
| *O. macintoshi* | E.10 | Belize | KM083854 | Present study |
| *O. macintoshi* | E.10 | Belize | KM083870 | Present study |
| *O. macintoshi* | E.10 | Belize | KM083875 | Present study |
| *O. macintoshi* | E.10 | Belize | KM083884 | Present study |
| *O. duplex* | E.11 | Belize | KM083847 | Present study |
| *O. duplex* | E.11 | Belize | KM083855 | Present study |
| *O. duplex* | E.11 | Belize | KM094175 | Present study |
| *O. duplex* | E.11 | Bocas del Toro | KM083850 | Present study |
| *O. duplex* | E.11 | Bocas del Toro | KM083856 | Present study |
| *O. duplex* | E.11 | Bocas del Toro | KM083857 | Present study |
| *O. duplex* | E.11 | Bocas del Toro | KM083878 | Present study |
| *O. duplex* | E.11 | Bocas del Toro | KM083885 | Present study |
| *O. duplex* | E.11 | Bocas del Toro | KM083889 | Present study |
| *O. macintoshi* | E.12 | Bocas del Toro | KM083842 | Present study |
| *O. macintoshi* | E.13 | Naos | KM083877 | Present study |
| *O. santacruzensis* | E.14 | USA, MA | AJ436913 | Thollesson, Norenburg 2003 |
| *O. parmula* | E.15 | Naos | KM083846 | Present study |
| *O. parmula* | E.15 | Naos | KM083859 | Present study |
| *O. parmula* | E.15 | Naos | KM083867 | Present study |
| *O. erneba* | E.16 | Naos | KM083869 | Present study |
| *O. erneba* | E.17 | Naos | KM083866 | Present study |
| *O. erneba* | E.18 | Bocas del Toro | KM083848 | Present study |
| *O. erneba* | E.18 | Bocas del Toro | KM083861 | Present study |
| *O. erneba* | E.18 | Bocas del Toro | KM083871 | Present study |
| *O. erneba* | E.18 | Bocas del Toro | KM083876 | Present study |
|  |  |  |  |  |
| ***Tetrastemma* spp.** |  |  |  |  |
| *T.vermiculus* | E.1 | Spain Med | AY791997 | Strand, Sundberg 2005 |
| *T.melanocephalum* | E.2 | Spain Med | AY791982 | Strand, Sundberg 2005 |
| *T.melanocephalum* | E.2 | Spain | AY791983 | Strand, Sundberg 2005 |
| *T.melanocephalum* | E.2 | Italy | AY791987 | Strand, Sundberg 2005 |
| *T.candidum* | E.3 | Spain Atl | AY791978 | Strand, Sundberg 2005 |
| *T.candidum* | E.4 | UK | AY791973 | Strand, Sundberg 2005 |
| *T.vermiculus* | E.4 | UK | AY791996 | Strand, Sundberg 2005 |
| *T.melanocephalum* | E.5 | Sweden | AY791988 | Strand, Sundberg 2005 |
| *T.melanocephalum* | E.5 | Sweden | AY791989 | Strand, Sundberg 2005 |
| *T.flavidum* | E.6 | UK | AY791977 | Strand, Sundberg 2005 |
| *T.candidum* | E.7 | Azores | AY791974 | Strand, Sundberg 2005 |
| *T.coronatum* | E.7 | Azores | AY791975 | Strand, Sundberg 2005 |
| *T.coronatum* | E.7 | Azores | AY791976 | Strand, Sundberg 2005 |
| *T.melanocephalum* | E.8 | UK | AY791986 | Strand, Sundberg 2005 |
| *T.melanocephalum* | E.9 | Spain | AY791984 | Strand, Sundberg 2005 |
| *T.peltatum* | E.10 | Italy | AY791992 | Strand, Sundberg 2005 |
| *T.longissimum* | E.11 | Azores | AY791981 | Strand, Sundberg 2005 |
| *T.robertianae* | E.12 | Sweden | AY791994 | Strand, Sundberg 2005 |
| *T.laminariae* | E.13 | Norway | AY791979 | Strand, Sundberg 2005 |
| *T.laminariae* | E.13 | Norway | AY791980 | Strand, Sundberg 2005 |
| *T.vermiculus* | E.14 | Italy | AY791995 | Strand, Sundberg 2005 |
| *T.peltatum* | E.15 | Italy | AY791993 | Strand, Sundberg 2005 |
| *T.peltatum* | E.16 | Italy | AY791990 | Strand, Sundberg 2005 |
| *T.peltatum* | E.16 | Italy | AY791991 | Strand, Sundberg 2005 |
| *T.albidum* | E.17 | USA, FL | EF157598 | Maslakova, Norenburg 2006 |
| *T.melanocephalum* | E.18 | Florida | AY791985 | Strand, Sundberg 2005 |
| *T.*sp.20 | E.19 | Naos | KM042064 | Present work |
| *T.*sp.2 | E.20 | Belize | KM042065 | Present work |
| *T.*sp.4 | E.21 | Bocas del Toro | KM042063 | Present work |
| *T.*sp.3 | E.22 | Bocas del Toro | KM042058 | Present work |
| *T.*sp.1 | E.23 | Belize | KM042057 | Present work |
| *T.*sp.1 | E.23 | Belize | KM042059 | Present work |
| *T.*sp.1 | E.23 | Belize | KM042061 | Present work |
| *T.*sp.3 | E.24 | Bocas del Toro | KM042060 | Present work |
| *T.*sp.5 | E.25 | Naos | KM042062 | Present work |
| *T.roseocephalum* | E.26 | Japan | AB725600 | Kajihara et al. 2012 |
| *T.roseocephalum* | E.26 | Japan | AB725601 | Kajihara et al. 2012 |
| *T.roseocephalum* | E.27 | Vietnam | AB725598 | Kajihara et al. 2012 |
| *T.pimaculatum* | E.28 | Russia | AB725597 | Kajihara et al. 2012 |
| *T.roseocephalum* | E.28 | China | AB725599 | Kajihara et al. 2012 |
|  |  |  |  |  |
